# Disparities in Biological Aging and Brain Health: Insights from Diverse Midlife Adults

**DOI:** 10.1002/alz70862_109971

**Published:** 2025-12-23

**Authors:** Indira C. Turney, Benjamin D. Huber, Calen P. Ryan, Justina F. Avila, Patrick J. Lao, Miguel Arce Rentería, Jessica Mazen, Daniel W. Belsky, Jennifer J. Manly, Adam Brickman

**Affiliations:** ^1^ National Institutes of Health / National Institute on Aging, Baltimore, MD USA; ^2^ Columbia University Irving Medical Center, New York, NY USA; ^3^ Mailman School of Public Health, Columbia University, New York, NY USA

## Abstract

**Background:**

Advanced biological is associated with cumulative physiological damage resulting from chronic exposure to environmental and social stressors. The Weathering Hypothesis posits that such exposure—especially in marginalized populations—accelerates biological aging, contributing to health disparities and elevated Alzheimer’s disease and related dementias (AD/ADRD) risk. Midlife is a pivotal period for brain aging, marked by structural changes such as increased white matter hyperintensity (WMH) volume and reduced cortical thickness (CT). However, the interplay among advanced biological aging, MRI‐based brain aging markers, and social determinants of health across racially/ethnically diverse populations remains poorly understood. Understanding these dynamics is essential for addressing disparities in brain health and AD/ADRD outcomes.

**Method:**

We examined the association between biological age acceleration (GrimAge) and ADRD‐related MRI measures (WMH volume and CT) in 681 racially/ethnically diverse adults aged 45–65 years from the Offspring Study of Racial and Ethnic Disparities in Alzheimer’s Disease. Linear regression models assessed the relationships between biological age acceleration and MRI outcomes, adjusting for age, sex, and estimated intracranial volume. Stratified analyses explored associations within racial/ethnic groups. Interaction terms tested the moderating effects of years of education and cardiovascular disease (CVD) burden on these associations.

**Result:**

In this diverse midlife cohort (66% women; 69% Latinx, 22% Black, 7.5% White; mean age=54±11 years; mean education=13.3 ±3.4 years), accelerated aging was significantly associated with increased WMH volume (β=0.014, 95%CI [0.003,0.025]), but not cortical thickness (β=0.562, 95%CI [0.003,0.025]). The relationship between biological age and WMH was amplified in participants with greater CVD burden (β=0.028, 95%CI [0.011,0.045]). Participants with higher education, Latinx, or White showed less biological age acceleration. Stratified analyses revealed that the association between biological age acceleration and greater WMH volume was marginally strongest in Black (β=0.013, 95%CI [‐0.008,0.033]) and Latinx participants (β=0.014, 95%CI [0.001,0.028]), compared with White participants.

**Conclusion:**

Accelerated biological aging is associated with increased WMH volume and may contribute to disparities in brain health, particularly among individuals with greater cardiovascular burden. These results highlight the need for targeted, multifaceted interventions that account for sociocultural and biological influences to reduce brain health disparities and promote equity in ADRD outcomes.

